# Supplementary material for: Human transcription factor protein interaction networks
Source: Nat Commun. 2022 Feb 9;13:766. doi: 10.1038/s41467-022-28341-5 (PMC8828895; doi:10.1038/s41467-022-28341-5)
Supplement: Supplementary file 1 — Supplementary Information [file 41467_2022_28341_MOESM1_ESM.pdf]

Supplementary Figures

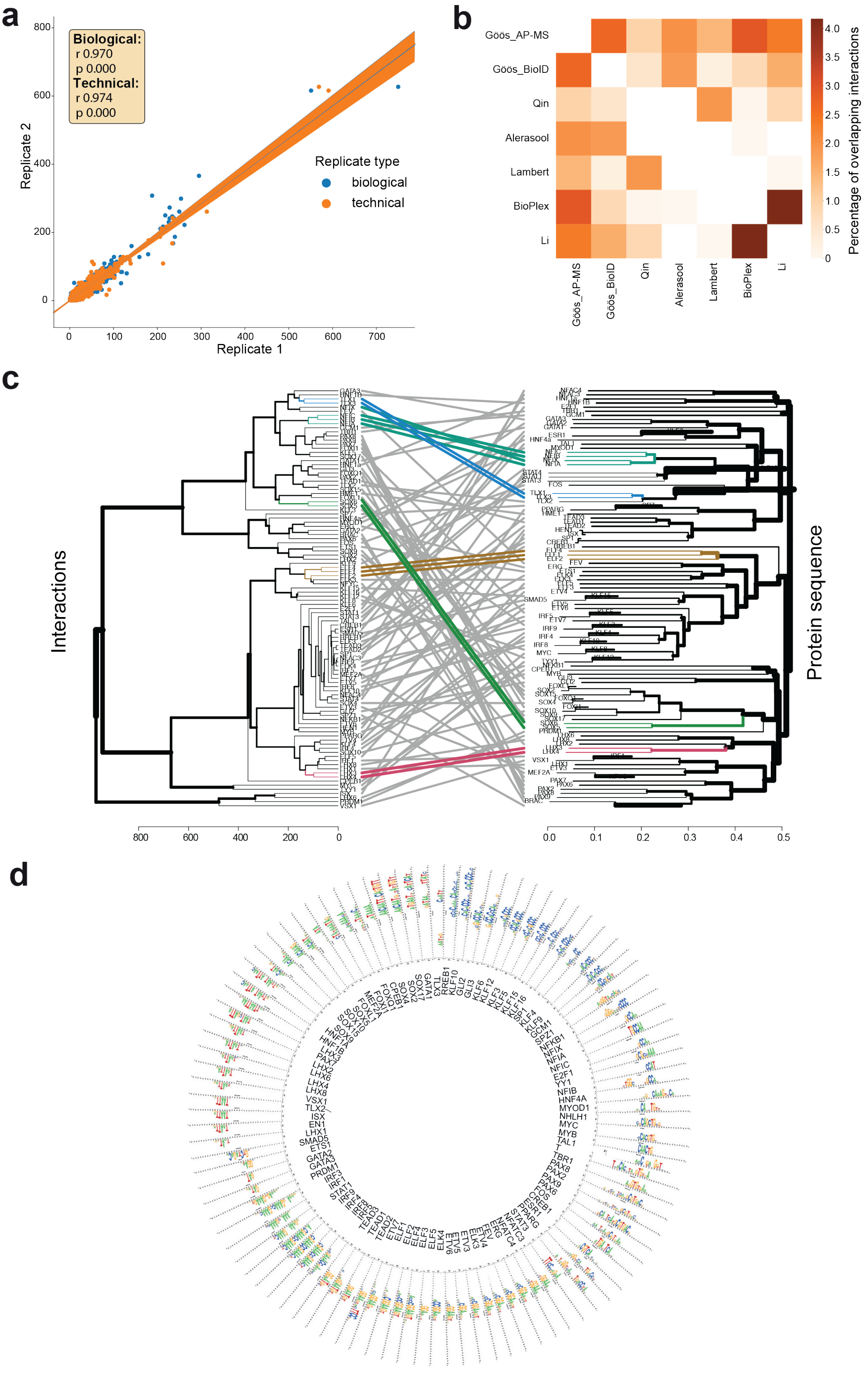

Supplementary Figure 1. DNA-binding motif alignment of studied TFs and its correlations to bait-prey clustering. a Correlation plots of biological (blue) and technical (orange) replicates performed with *seaborn and scipy* python packages. Correlation coefficient r for each of the replicate types is shown in the box in top left corner. **b** Heatmap of bait-prey interaction overlap between data presented in this paper (Göös_APMS, Göös_BioID), BioPlex, and four other large-scale transcription factor interactome studies. Values shown refer to the percentage of overlapping interactions, and sets were not compared against themselves to preserve contrast. **c** Comparison of TF interaction clustering and full-length amino acid sequence alignment by *dendexted* R package. TF groups with significant correlation between PPI clustering and full-length amino acid sequence are colour coded. **d** TFs were aligned by DNA-binding motifs (JASPAR, HT-SELEX and ENCODE) using RSAT. Source data of the figure are provided as a Source Data file.

a

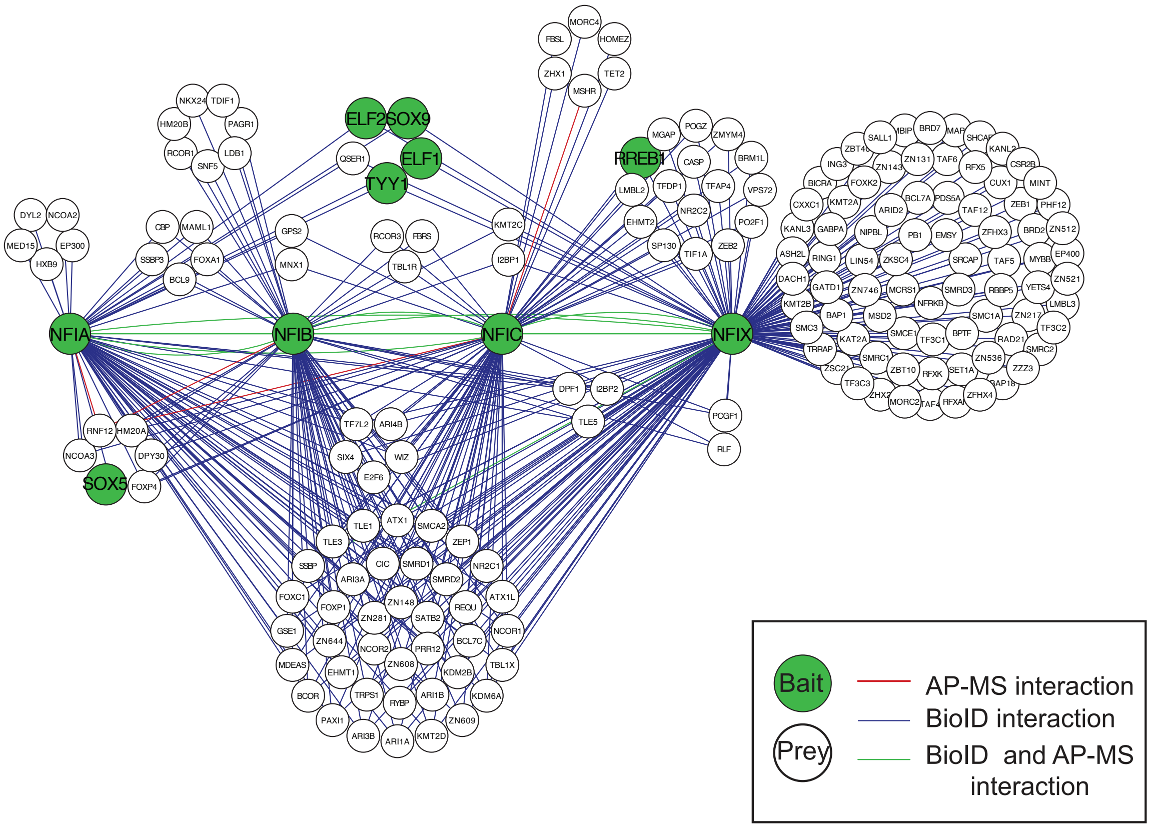
NFIA
NFIB
NFIC
NFIX
ELF2
SOX9
ELF1
YY1
RREB1
SOX5
Bait
Prey
AP-MS interaction
BioID interaction
BioID and AP-MS interaction

b

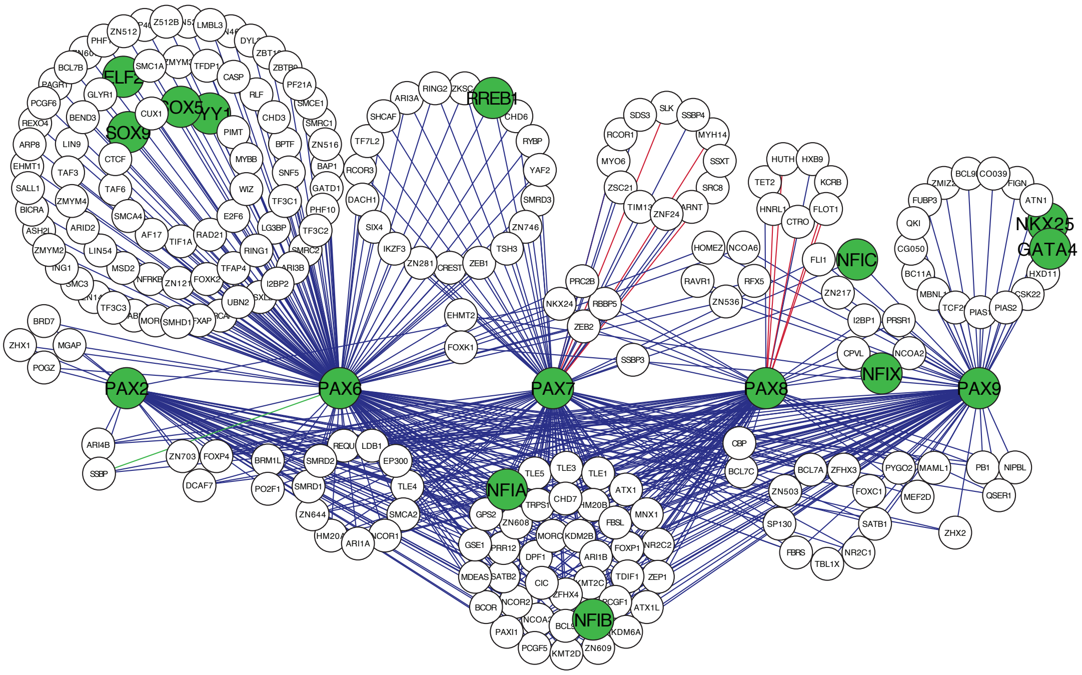
PAX2
PAX6
PAX7
PAX8
PAX9
NFIA
NFIB
NFIC
NFIX
RREB1
SOX9
ELF2
YY1
NKX25
GATA4

Supplementary Figure 2. Protein-protein interactions of studied NFIs and PAXs. a AP-MS and BioID interactions of NFI family TFs. **b** AP-MS and BioID interactions of five PAX -TFs studied. Green nodes indicate the baits studied and the white nodes the interacting prey proteins. BioID interactions are shown with blue edges, AP-MS interactions with red edges and interactions detected in both methods with green edges. Source data of the figure are provided as a Source Data file.

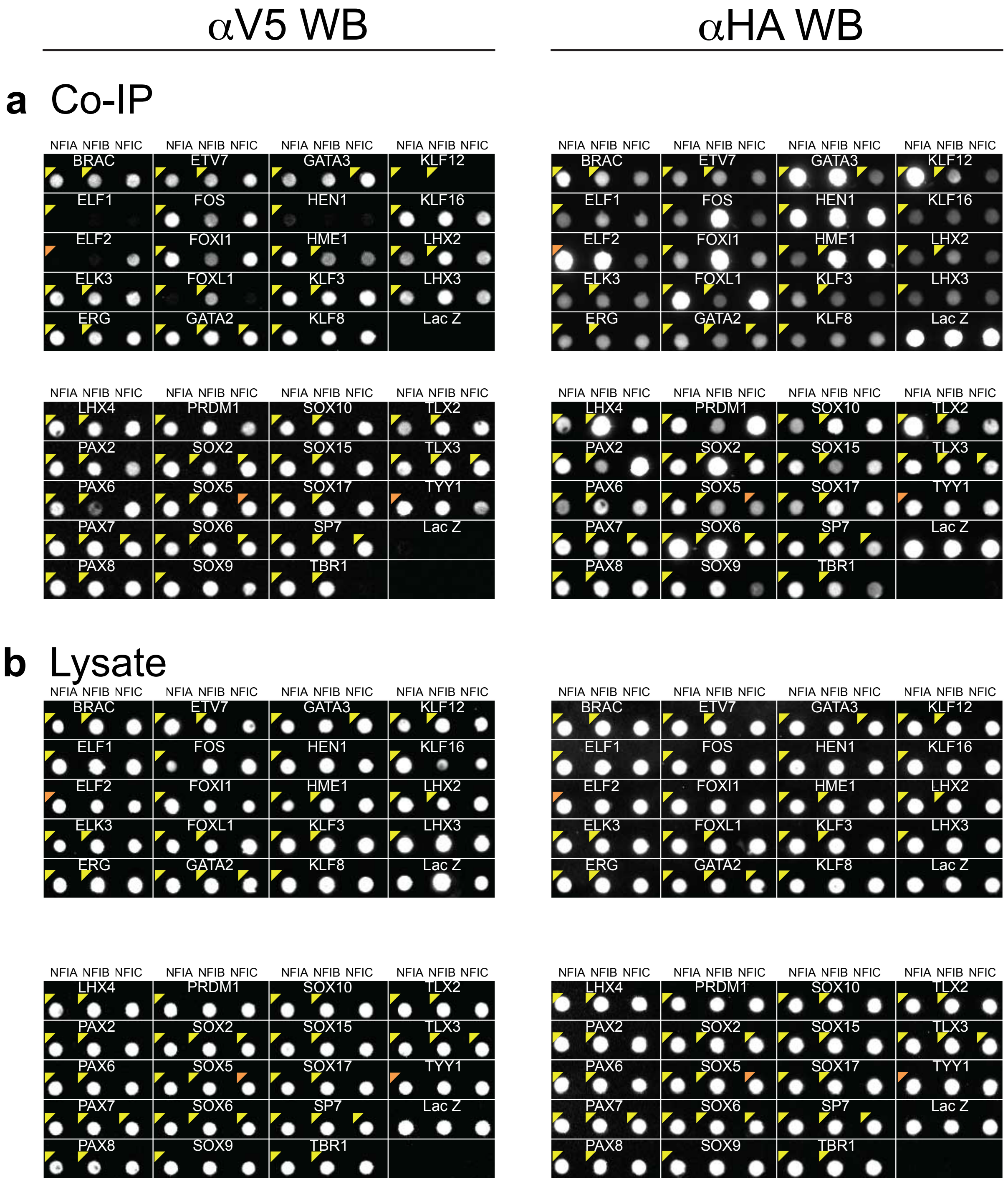
αV5 WB
αHA WB
a Co-IP
NFIA NFIB NFIC
BRAC
ETV7
GATA3
KLF12
ELF1
FOS
HEN1
KLF16
ELF2
FOXI1
HME1
LHX2
ELK3
FOXL1
KLF3
LHX3
ERG
GATA2
KLF8
Lac Z
LHX4
PRDM1
SOX10
TLX2
PAX2
SOX2
SOX15
TLX3
PAX6
SOX5
SOX17
TYY1
PAX7
SOX6
SP7
Lac Z
PAX8
SOX9
TBR1
b Lysate

Supplementary Figure 3. Validation of NFI family TF-TF interactions, detected by mass spectrometry, using co-immunoprecipitation and dot blotting. HEK293 cells were co-transfected with one of the corresponding strep-HA-tagged NFIA, NFIB or NFIC and one of the 37 TFs tagged with V5-epitope. These 37 TFs were detected as high-confidence interactors for minimally with one of the NFI family members either as a prey or bait in our interaction analyses (BioID or AP-MS). **a** Dot blot detection, with anti-HA and anti-V5 antibodies, of the samples after Co-IP. **b** Dot blot detection, with HA and V5 antibodies, of the lysate. The pair-wise combinations which validated the high-confidence interactions detected with BioID or AP-MS are highlighted with yellow. Additionally, novel interactions of TFs with NFI family members are shown with orange. V5-tagged Lac Z was used as a negative control. Source data of the figure are provided as a Source Data file.

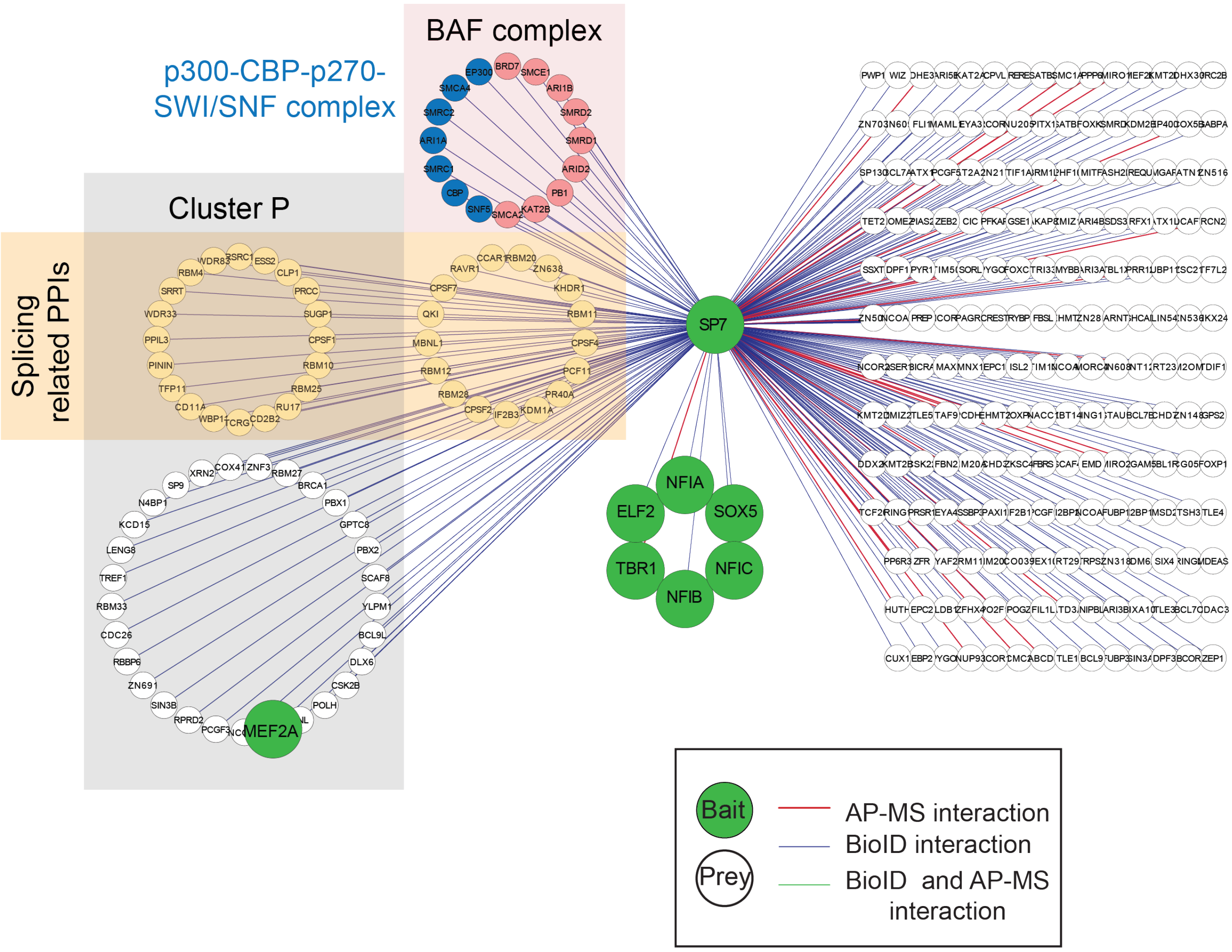

Supplementary Figure 4. In addition to splicing-related interactions in Clusters P and Q, we found that SP7 interacted with EP300 (p300) along with other members of the p300-CBP-p270-SWI/SNF and BAF complexes. BioID interactions are shown with blue edges, AP-MS interactions with red edges and interactions detected in both methods with green edges. Source data of the figure are provided as a Source Data file.
