# Supplementary material for: Human transcription factor protein interaction networks
Source: Nat Commun. 2022 Feb 9;13:766. doi: 10.1038/s41467-022-28341-5 (PMC8828895; doi:10.1038/s41467-022-28341-5)
Supplement: Supplementary file 2 — Reporting Summary [file 41467_2022_28341_MOESM2_ESM.pdf]

nature research

Corresponding author(s): Markku Varjosalo

Last updated by author(s): Jan 13, 2022

Reporting Summary

Nature Research wishes to improve the reproducibility of the work that we publish. This form provides structure for consistency and transparency in reporting. For further information on Nature Research policies, see our Editorial Policies and the Editorial Policy Checklist.

Statistics

For all statistical analyses, confirm that the following items are present in the figure legend, table legend, main text, or Methods section.

n/a	Confirmed	
☐	☒	The exact sample size (*n*) for each experimental group/condition, given as a discrete number and unit of measurement
☐	☒	A statement on whether measurements were taken from distinct samples or whether the same sample was measured repeatedly
☐	☒	The statistical test(s) used AND whether they are one- or two-sided<br>*Only common tests should be described solely by name; describe more complex techniques in the Methods section.*
☒	☐	A description of all covariates tested
☒	☐	A description of any assumptions or corrections, such as tests of normality and adjustment for multiple comparisons
☐	☒	A full description of the statistical parameters including central tendency (e.g. means) or other basic estimates (e.g. regression coefficient) AND variation (e.g. standard deviation) or associated estimates of uncertainty (e.g. confidence intervals)
☐	☒	For null hypothesis testing, the test statistic (e.g. *F*, *t*, *r*) with confidence intervals, effect sizes, degrees of freedom and *P* value noted<br>*Give P values as exact values whenever suitable.*
☒	☐	For Bayesian analysis, information on the choice of priors and Markov chain Monte Carlo settings
☒	☐	For hierarchical and complex designs, identification of the appropriate level for tests and full reporting of outcomes
☐	☒	Estimates of effect sizes (e.g. Cohen's *d*, Pearson's *r*), indicating how they were calculated

Our web collection on statistics for biologists contains articles on many of the points above.

Software and code

Policy information about availability of computer code

Data collection	Data was collected in the study using commercial Thermo Scientific™ Xcalibur™ Software. Version 2.7.0
Data analysis	CRAPome 2.0, Mellacheruvu et al., 2013 http://www.crapome.org/<br>Cytoscape version 3.8.2, Shannon et al., 2003 http://www.cytoscape.org/<br>DAVID Bioinformatics Resources, National Institute of Allergy and Infectious Diseases (NIAID), NIH https://david.ncifcrf.gov/home.jsp<br>Gene Ontology analysis tool DAVID, Bioinformatics Resources 6.8 National Institute of Allergy and Infectious Diseases (NIAID), NIH https://david.ncifcrf.gov/home.jsp<br>Hierarchial clustering tool Pro-Hits-viz, Gigras lab, Department of Molecular Genetics, University of Toronto, Toronto, Ontario, Canada https://prohits-viz.lunenfeld.ca<br>Interaction network analysis tool: PINA v2.0, Cowley et al., 2012 http://cbg.garvan.unsw.edu.au/pina/<br>STRING: functional protein association network, version 11, Szklarczyk et al. 2019, https://string-db.org<br>IntAct Molecular Interaction Database, Orchard et al 2013,https://www.ebi.ac.uk/intact/home<br>BrioGRID, Database of Protein, Genetic and Chemical Interactions, version 4.4, Oughtred et al. 2021, https://thebiogrid.org<br>Mammalian protein complex resource: CORUM Institute of Bioinformatics and Systems Biology, Giurgiu et al. 2019, Helmholtz Zentrum München, http://mips.helmholtz-muenchen.de/corum/,<br>Proteome Discoverer version 1.4, Thermo Scientific, https://www.Thermo Fisher.com/fi/en/home.html<br>SAINTexpress version 3.1.0, Choi et al., 2011, http://saint-apms.sourceforge.net/Main.html<br>Uniprot , release 2018_01; 20,192 entries, https://www.uniprot.org/https:/<br>Xcalibur version 2.7.0 Thermo Scientific https://www.Thermo Fisher.com/fi/en/home.html<br>The dendexted R package (https://www.datanovia.com/en/lessons/comparing-cluster-dendrograms-in-r/), version 1.14.0<br>JASPAR database (7th release), Mathelier et al. 2016, https://jaspar.genereg.net<br>HT-SELEX database, Jagannathan et al 2006, https://ccg.epfl.ch/htpselex/

ENCODE, https://maayanlab.cloud/Harmonizome/dataset/ENCODE+Transcription+Factor+Targets
Regulatory Sequence Analysis Tools (RSAT), http://rsat.sb-roscoff.fr, Nguyen et al 2018.
Phyton SciPy (include scipy.stats) package (version 1.71)
Phyton seaborn package (version 0.11.2)

For manuscripts utilizing custom algorithms or software that are central to the research but not yet described in published literature, software must be made available to editors and reviewers. We strongly encourage code deposition in a community repository (e.g. GitHub). See the Nature Research guidelines for submitting code & software for further information.

Data

Policy information about availability of data

All manuscripts must include a data availability statement. This statement should provide the following information, where applicable:

- Accession codes, unique identifiers, or web links for publicly available datasets
- A list of figures that have associated raw data
- A description of any restrictions on data availability

The source data of the figures are provided with this paper as a separate Excel sheet.

The MS peptide raw data from the MS runs have been deposited in the Massive database (http://massive.ucsd.edu/ProteoSAFe/status.jsp?task=0bfbe238f2ab4bd1a12fec75e4f6c67e) under accession number MSV000086891.

The protein interactions from this publication have been submitted to the IMEx (http://www.imexconsortium.org) consortium through IntAct110 and assigned the identifier IM-28767.

Filtered protein–protein interactions are also available in Table S1.

Following databases were used in data analysis:

Uniprot (release 2018_01; 20,192 entries, https://www.uniprot.org)
CRAPome 2.0 (http://www.crapome.org/), PINA Interaction network analysis tool (version 2.0, http://cbg.garvan.unsw.edu.au/pina/)
STRING: functional protein association network database, version 11, (https://string-db.org)
IntAct Molecular Interaction Database (https://www.ebi.ac.uk/intact/home), downloaded 30.4.2021
BrioGRID, Database of Protein, Genetic and Chemical Interactions (version 4.4, https://thebiogrid.org)
Mammalian protein complex resource: CORUM Institute of Bioinformatics and Systems Biology (http://mips.helmholtz-muenchen.de/corum/)
JASPAR database (7th release, https://jaspar.genereg.net)
HT-SELEX database (https://ccg.epfl.ch/htpselex/)
ENCODE database, (https://maayanlab.cloud/Harmonizome/dataset/ENCODE+Transcription+Factor+Targets)

Field-specific reporting

Please select the one below that is the best fit for your research. If you are not sure, read the appropriate sections before making your selection.

[x] Life sciences [ ] Behavioural & social sciences [ ] Ecological, evolutionary & environmental sciences

For a reference copy of the document with all sections, see nature.com/documents/nr-reporting-summary-flat.pdf

Life sciences study design

All studies must disclose on these points even when the disclosure is negative.

Sample size	Sufficient sample sizes were chosen for each experiment to determine whether the outcome was statistically significant. For cell line experiments minimal number replicates for each experiments was two or more
Data exclusions	No data was excluded from the analysis
Replication	TFs were analyzed in two biological replicates and, as the correlation between the technical and biological replicates were excellent (Supplementary Fig. 1a), either in one or two technical replicates. TF activity was assessed by luciferase assays in three replicates. Co-immunoprecipitation-dot plot analysis was done without replication.
Randomization	Different samples (different baits or different analysis method) were allocated to MS analysis in a random order. The reporter assay groups were in random position order on assay plates.
Blinding	The generated cell lines and MS-samples were labeled by running number labels in analysis, not by the identity (gene/protein name) of the

TFs. The identity of TFs was opened when the data analysis began.

Reporting for specific materials, systems and methods

We require information from authors about some types of materials, experimental systems and methods used in many studies. Here, indicate whether each material, system or method listed is relevant to your study. If you are not sure if a list item applies to your research, read the appropriate section before selecting a response.

Materials & experimental systems

n/a	Involved in the study
☐	☒ Antibodies
☐	☒ Eukaryotic cell lines
☒	☐ Palaeontology and archaeology
☒	☐ Animals and other organisms
☒	☐ Human research participants
☒	☐ Clinical data
☒	☐ Dual use research of concern

Methods

n/a	Involved in the study
☒	☐ ChIP-seq
☒	☐ Flow cytometry
☒	☐ MRI-based neuroimaging

Antibodies

Antibodies used

Anti-NFIA, Abcam, Cat# ab228897, 1:1000
Anti-Rabbit, Dako, Cat# P0448, 1:1500
Anti-V5 antibody, ThermoFisher, Cat# R960-25, 1:5000
Goat anti-mouse IgG H&L (HRP), Abcam, Cat# 97023, 1:2000
Anti-HA, BioLegend, Cat# PRC-101C, 1:2000
Anti-Rabbit IgG H&L (HRP), Abcam, Cat# ab205718, 1:2000

Validation

Antibodies used in this study are commercially available and used in more than 100 published articles. In our studies we used negative and positive controls for validation of the antibodies prior or during their use.

Anti-NFIA, Abcam, Cat# ab228897 has been also used by Zhu Z et al. (MiRNA-671-5p Promotes prostate cancer development and metastasis by targeting NFIA/CRYAB axis. Cell Death Dis 11:949 (2020)) and the vendor has Abpromise guarantee that cover the use of this antibody for western blot (https://www.abcam.com/ctfnfia-antibody-ab228897.html?productWallTab=ShowAll).

Anti-Rabbit, Dako, Cat# P0448, has been widely used with more than 1200 citations (https://www.citeab.com/antibodies/3288347-p0448-goat-anti-rabbit-immunoglobulins-hrp-affinity). It also has CE-IVD certificate (https://www.agilent.com/store/en_US/Prod-P044801-2/P044801-2).

Anti-V5 antibody, ThermoFisher, Cat# R960-25, has references to more than 840 publications in manufactures webpage (https://www.thermofisher.com/antibody/product/V5-Tag-Antibody-Monoclonal/R960-25). They also state in their webpage that "This Antibody was verified by Relative expression to ensure that the antibody binds to the antigen stated."

Goat anti-mouse IgG H&L (HRP), Abcam, Cat# 97023, has references to more than 240 publications in manufactures webpage (https://www.abcam.com/goat-mouse-igg-hl-hrp-ab97023.html). It has also manufactures Abpromise guarantee that covers the use of ab97023 in western blot. The specificity is stated in webpage as following: "By immunoelectrophoresis and ELISA this antibody reacts specifically with Mouse IgG and with light chains common to other Mouse immunoglobulins. No antibody was detected against non immunoglobulin serum proteins."

Anti-HA, BioLegend, CAT# PRB-101C, has been used also by Trautz B, et al. 2016. J Virol. 90(23):10915-10927; Zhu Z, et al. 2016. J Virol. 90(24):11106-11121; Villeneuve J, et al. 2017. Mol Biol Cell. 28(1):141-151; Ting Y, et al. 2017. J Biol Chem. 292(2):585-596; Li J, et al. 2021. Med (N Y). 0.152083333 and Zeng J, et al. 2020. Cell Stem Cell. 27(4):618-632.e9. Manufacture says in their webpage (https://www.biolegend.com/fr-ch/products/anti-ha-tag-antibody-11070) that "This antibody is effective in immunoblotting (WB) and immunoprecipitation (IP) of tagged proteins."

Anti-Rabbit IgG H&L (HRP), Abcam, Cat# ab205718, has been widely used with 1670 citations on manufactures web page (https://www.abcam.com/goat-rabbit-igg-hl-hrp-ab205718.html). The specificity is described as follows: "The antibody used for conjugation reacts with rabbit immunoglobulins of all classes. Crossreactions as determined by ELISA for the unconjugated antibody (ab182016): Mouse IgG, rat IgG, and chicken IgY, less than 2%. Human IgG, less than 7%."

Eukaryotic cell lines

Policy information about cell lines

Cell line source(s)

Human: HEK 293 cell line, ATCC, Cat# ATCC CRL-1573™

	Human: HEK Flp-In T-REx 293 cell line, Invitrogen, Cat# R78007
Authentication	Both the cell line was obtained directly from commercial sources; additionally only low passage cells (passage number <10) were used for experiments. Manufacturers are known to follow the authentication of cells lines batches regularly and certificates of authentication were provided with the cells.
Mycoplasma contamination	cell lines tested negative for mycoplasma
Commonly misidentified lines (See ICLAC register)	no commonly misidentified cell lines used
