# Supplementary material for: Human transcription factor protein interaction networks
Source: Nat Commun. 2022 Feb 9;13:766. doi: 10.1038/s41467-022-28341-5 (PMC8828895; doi:10.1038/s41467-022-28341-5)
Supplement: Supplementary file 10 — Description of Additional Supplementary Files [file 41467_2022_28341_MOESM10_ESM.pdf]

Title: Supplementary Data 1.
Description: 1A109 selected human transcription factors for the protein-protein interaction analysis using AP-MS and BioID- methods.

1B. High-confidence human TF protein-protein interactions (PPIs) identified with BioID-method.

1C. High-confidence human TF protein-protein interactions (PPIs) identified with AP-MS-method.

Title: Supplementary Data 2.
Description: Subcellular localization data of BioID preys.

Title: Supplementary Data 3.
Description: Summary protein-protein interactions (PPIs) for each TF family identified by BioID-method.

Title: Supplementary Data 4.
Description: Gene Ontology Biological Process (GO-BP) enrichment analyses of identified prey proteins by DAVID Bioinformatics Resources.

Title: Supplementary Data 5.
Description: General transcription factors, SAGA complex members and Poll-II components interacting with studies TFs.

Title: Supplementary Data 6.
Description: The TF prey-prey correlation analysis using ProHits-Viz revealed 17 biological clusters
